# Red narrow-linewidth lasing and frequency comb from gain-switched self-injection-locked Fabry–Pérot laser diode

**DOI:** 10.1038/s41598-023-36229-7

**Published:** 2023-06-17

**Authors:** Artem E. Shitikov, Ramzil R. Galiev, Kirill N. Min’kov, Nikita M. Kondratiev, Steevy J. Cordette, Valery E. Lobanov, Igor A. Bilenko

**Affiliations:** 1 Russian Quantum Center, Skolkovo, Moscow, 143025 Russia; 2 Directed Energy Research Centre, Technology Innovation Institute, Abu Dhabi, United Arab Emirates; 3 Faculty of Physics, Lomonosov Moscow State University, Moscow, 119991 Russia

**Keywords:** Diode lasers, Frequency combs, Photonic devices

## Abstract

Narrow-linewidth lasers are in extensive demand for numerous cutting-edge applications. Such lasers operating at the visible range are of particular interest. Self-injection locking of a laser diode frequency to a high-Q whispering gallery mode is an effective and universal way to achieve superior laser performance. We demonstrate ultranarrow lasing with less than 10 Hz instantaneous linewidth for 20 $\mu$s averaging time at 638 nm using a Fabry–Pérot laser diode locked to a crystalline MgF$_2$ microresonator. The linewidth measured with a $\beta$-separation line technique that characterizes 10 ms stability is as low as 1.4 kHz. Output power exceeds 80 mW. Demonstrated results are among the best for visible-range lasers in terms of linewidth combined with solid output power. We additionally report the first demonstration of a gain-switched regime for such stabilized Fabry–Pérot laser diode showing a high-contrast visible frequency comb generation. Tunable linespacing from 10 MHz to 3.8 GHz is observed. We demonstrated that the beatnote between the lines has sub-Hz linewidth and experiences spectral purification in the self-injection locking regime. This result might be of special importance for spectroscopy in the visible range.

## Introduction

Narrow-linewidth lasers are in increasing demand for numerous cutting-edge technologies^1–5^. They open up new possibilities in advanced areas such as position/navigation/timing systems, coherent communications, precision spectroscopy, optical ranging, frequency synthesizers, and precision measurements. Self-injection locking (SIL) of a laser diode frequency to an eigenfrequency of a whispering gallery mode microresonator has already proved its effectiveness for manufacturing ultrastable compact laser sources with unprecedented narrow lines^6^. A couple of decades ago, SIL was demonstrated for the first time in optics for the stabilization of red lasers to kHz-linewidth^7,8^. Nowadays applicability and high efficiency of this approach have been demonstrated for a vast range of wavelengths from UV to mid-IR^9–14^. Nevertheless, the best results were obtained for telecommunication wavelengths. Latterly the thermo-refractive noise limit has become a limiting factor for stabilized lasers^15,16^. Hertz-scale linewidths limited by thermo-refractive noise were demonstrated^10,17,18^. One of the ways to overcome the thermo-refractive noise is by using large spiral resonators^19,20^. SIL lasers demonstrate their best stability for short averaging time ($\approx$ ms). At a longer timescale, thermal drift of the resonator usually gives the main contribution to the instability. While the Pound–Drever–Hall (PDH) locking technique demonstrated excellent experimental results regarding linewidth and residual phase noise^20–22^, SIL remains the most straightway and robust approach both for laboratory and consumer devices.

The visible spectral range is of paramount importance for many up-to-date applications^23^. This range is very interesting for biosensing since strong absorption in many samples is observed in near-IR. Also, molecules often undergo strong absorption lines in the visible range, so narrow-linewidth lasers are required as spectrum study instruments. In addition, the atomic clocks, e.g., based on Rb and Cs transition lines, require laser sources with narrow linewidth and a certain frequency in the visible range. Among the other important applications that need narrow-linewidth visible lasers, one can mention quantum photonics, including quantum sensing and communications^24,25^, high-capacity communication systems^26^, laser-based position/navigation/timing systems^27,28^, laser-based displays^29^ inclusive of displays for augmented reality and virtual reality^30^.

However, for shorter wavelengths, the SIL-based approach can be limited by the high Rayleigh scattering as it reduces the Q-factor of the microresonator, decreasing the stabilization coefficient^31,32^.

SIL Fabry–Pérot laser diode operating at a wavelength of 446.5 nm with a linewidth of less than 1 MHz^33^ and sub-100 kHz UV laser at 370 nm^34^ have been demonstrated with crystalline microresonators so far. Recent advances in integrated photonics allowed to achieve SIL for the ultraviolet band, but it is still limited to several kHz Lorentzian and more than a hundred kHz Gaussian linewidth^5,35^. Promising results were demonstrated using second harmonic generation with a SIL laser source, reaching a linewidth of tens of kHz at 780 nm^36^. Another effective way for achieving a stable laser is based on the stimulated Brillouin scattering. The instantaneous laser linewidths of 1.6 Hz at 1550 nm^37^ and 269 Hz at 674 nm^38^ in silicon nitride on-chip microresonator were demonstrated with this technique.

Microresonator-based stabilization allows laser operation in the gain-switching (GS) regime^39,40^. Gain switching via modulation of a laser diode operating current through a bias tee circuit allows for the generation of a frequency comb with line spacing defined by the microwave modulation frequency^41–43^. This frequency comb generation benefits from unprecedented flexibility and simplicity, which are useful for spectroscopy^44^ and high-capacity communication^45^. Due to the combination of SIL and GS, all generated GS comb lines can be narrowed to the sub-kHz level^40^. In the SIL GS regime, the mode spacing can be arbitrarily small—a versatility has been hard to achieve with micro-combs generated by four-wave-mixing in Kerr microresonators^46^. GS combs have relatively narrow spectra of about several nanometers wide, but they can be some alternative for Kerr micro-combs in the visible range. Kerr micro-combs lines above $\sim \, 700$ nm are difficult to obtain because of strong normal dispersion in most materials at shorter wavelengths and also have narrow spectra compared to Kerr combs at telecom bandwidth^47–49^. Generation of Kerr combs in the normal group-velocity dispersion (GVD) regime also requires a complicated setup, such as a multiresonator circumference design for dispersion control^50,51^, high-frequency pump modulation systems^52,53^ or bichromatic pump^54^. Thus, gain-switched visible frequency combs can be useful for many important applications, such as high bit-rate visible communication^55^, optical coherence tomography systems^56,57^, and biosensing.

In this work, we demonstrate a narrow-linewidth lasing at 638 nm from a visible Fabry–Pérot laser diode self-injection locked to a high-Q MgF$_2$ microresonator. It was shown that even in the visible range, SIL leads to outstanding laser linewidth narrowing and stabilization. For accurate characterization of the spectral properties of such a laser source, the heterodyne method was applied. The beatnote signal was collected, and frequency noise spectral density at a frequency offset was analysed. The instantaneous linewidth of the stabilized laser is less than 10 Hz. Also, a $\beta$-separation line technique^58^ was used for characterizing the long-term stability of the laser, resulting in a linewidth of less than 1.4 kHz. These values may be considered as a linewidth of the laser at 20 $\upmu$s averaging time and 10 ms consequently. The direct observation of the beatnote gives us the linewidth of less than 850 Hz for a measurement time of 12.5 ms. In our case, relatively low confinement of the beam with the WGM ($\approx$ 15 %) leads to a low reduction of the output power during self-injection locking—before stabilization, it was 85 mW and 80 mW after. In terms of linewidth and output power, these results are among the best for laser diodes in the visible range. A gain-switching technique was implemented to the stabilized laser diode, and the generation of a high-contrast frequency comb with narrow lines and tunable line spacing was demonstrated. This result expands the area of SIL gain-switched laser diodes applications both for the visible range and Fabry–Pérot diode type. Relatively high power and superior frequency stability address the challenges of biosensing and tunability of comb lines in a gain-switch regime allowing for extremely fine tuning to reach atomic clock transitions.

## Results and discussion

### Experiment concept

**Figure 1:**
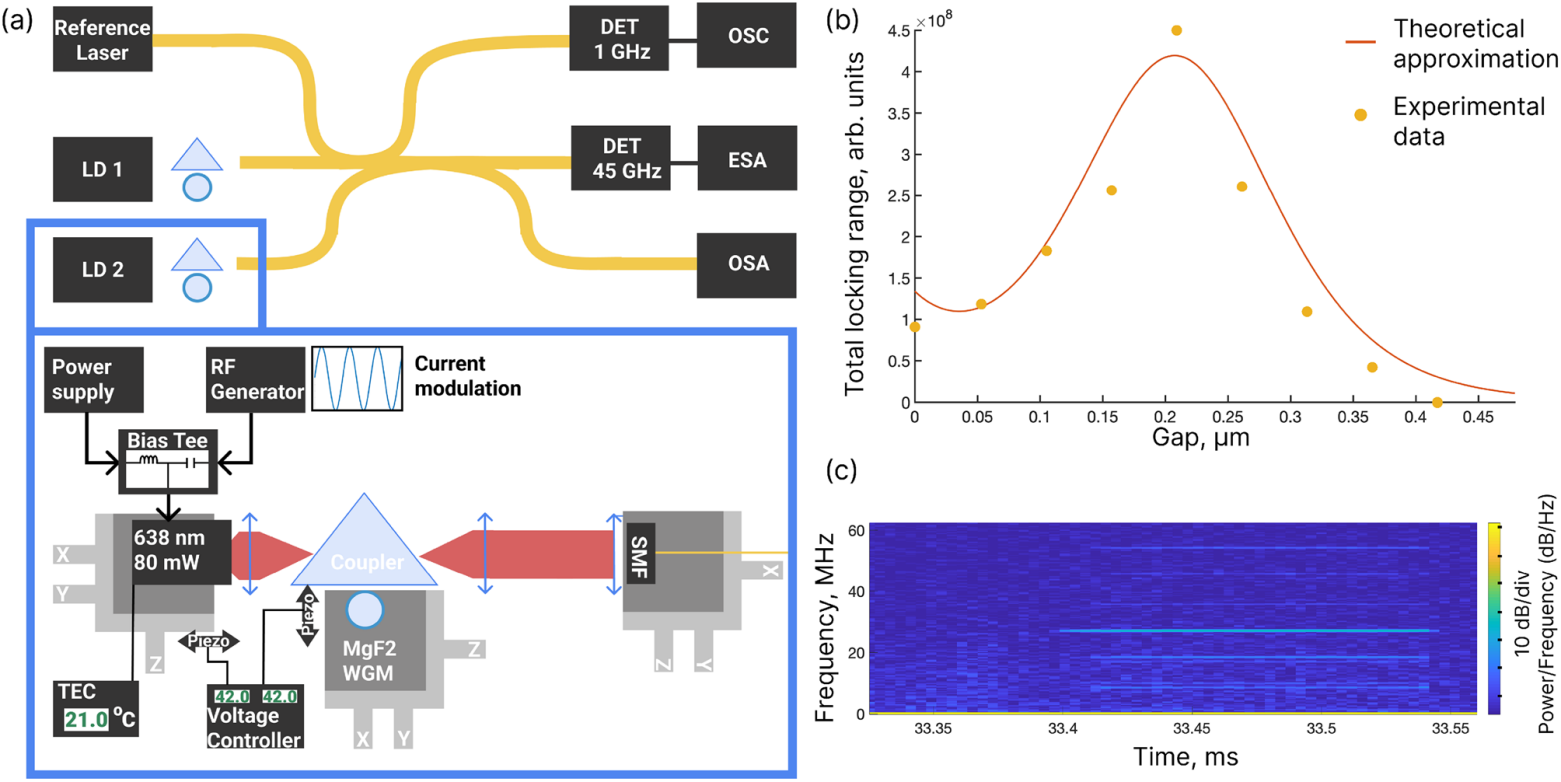
(**a**) Sketch of the experimental setup. Two 638 nm Fabry–Pérot laser diodes are stabilized independently by high-Q WGM microresonators made of MgF$_2$. Transmitted light is coupled into the single-mode optical fiber and transferred to measuring equipment: oscilloscope (OSC) through a 1 GHz bandwidth detector, electrical spectrum analyser (ESA) through a 45 GHz bandwidth detector, and optical spectrum analyser (OSA). There is an option to beat the SIL lasers with a reference laser for heterodyne detection. In the blue frame the detailed scheme of the SIL laser is presented. Laser diode current can be modulated through a bias tee circuit to achieve a gain switching regime. Laser diodes have thermo-electric control (TEC). The distances between the laser diode and microresonator and coupling prism and microresonator are precisely controlled with piezo-stages. (**b**) Dependence of the locking range, measured in the experiment, on the distance between the coupling element and the microresonator. Yellow dots are experimental data, and the orange line is the approximation for internal $Q = (2.5\pm 0.5)\times 10^8$. (**c**) Spectrogram of the self-injection locked state of the gain-switching comb. Initially, the SIL regime is realized, and after 0.05 ms the sidebands appear. It can be seen that the lines of the comb are narrow.

An experimental setup is based on Fabry–Pérot laser diodes operating at 638 nm with up to 150 mW output power coupled to high-Q bulk MgF$_2$ microresonators. The temperatures of the diodes are stabilized. There are two lasers self-injection locked to high-Q microresonators made of magnesium fluoride. The operating current for one of them is provided through a bias tee circuit. Whispering gallery modes are excited with a prism coupler. The gap between the coupler and microresonator, as well as the distance from the laser to the coupler, are controlled with piezo stages. Detailed information about the setup is presented in Fig. 1a inside the blue frame. The transmitted light from each laser is coupled to a single-mode optical fiber SM600. Then the light from both of the SIL lasers and a reference laser are combined into a single fiber and split into an optical spectrum analyzer (OSA), electrical spectrum analyzer (ESA), and oscilloscope (OSC) (see the scheme of the experiment in Fig. 1a). To reduce acoustic and temperature fluctuations, as well as reduce consequent frequency deviations, we covered the setups with isolation boxes. A 1 GHz bandwidth photodetector is used with the oscilloscope, and a 45 GHz bandwidth photodetector with the ESA. The used ESA has a bandwidth of 26 GHz and provides the option of quadrature analysis.

For the experimental study of a gain-switching regime, we use an approach based on the laser current modulation through a bias tee circuit to observe the gain-switch frequency combs. To characterize the stability of such a source precisely, the laser under study is mixed with an identical laser diode self-injection locked to a similar microresonator. To measure the spectra of the gain-switched frequency combs, a tunable reference laser is used for heterodyning, providing enough flexibility in observation.

The experiment is divided into three stages: preliminary measurements characterizing the system (Fig. 1b), linewidth measurements in the self-injection locking regime, gain-switching frequency combs observation, and characterization. In the first stage, we measure the Q-factors of microresonators with a 1550 nm laser with an isolator and measure the basic characteristics of SIL at 638 nm. These characteristics are output powers, Q-factors (by measuring the dependence of the locking range from the gap between a microresonator and a coupler^12^), and locking wavelengths. Then at the second stage, we obtain simultaneous SIL regime of both of the Fabry–Pérot diode lasers to corresponding microresonators and measure the frequency noise of the beatnote of the two SIL laser diodes. Finally, in the third stage, we use a bias tee element to obtain a gain-switching frequency comb in one of the lasers and study comb formation dynamics via the spectrogram method^59^ (see Fig. 1c) and the characteristics of generated signals, including tunability.

### Microresonator characterization

In our experiments, we use four different MgF$_2$ microresonators with diameters of 3, 4, 6, and 7 mm. Their Q-factors are measured at 1550 nm with a narrow-linewidth laser with isolator using ringdown technique^60^, providing internal Q-factors from $5\times 10^8$ to $2\times 10^9$. If we assume that the Rayleigh scattering is a dominant mechanism of losses, it may give us an estimation that the Q-factors at 638 nm are about $10^8$. We conduct measurements of the Q-factor at 638 nm using an approach based on locking range dependence on the gap between the microresonator and the coupler^12^ and obtain good agreement. In Fig. 1b, the locking range width dependence on the gap between the microresonator of 4 mm diameter and the coupling prism is shown. The approximation of the experimental data gives us internal $Q = (2.5\pm 0.5)\times 10^8$ and vertical index of the mode $p = 0$^61^. We work in a critical coupling regime when coupling losses are equal to internal losses. The decay rate determined by the coupling loss can be estimated as 3.8 MHz.

### Self-injection locked laser characterization

**Figure 2:**
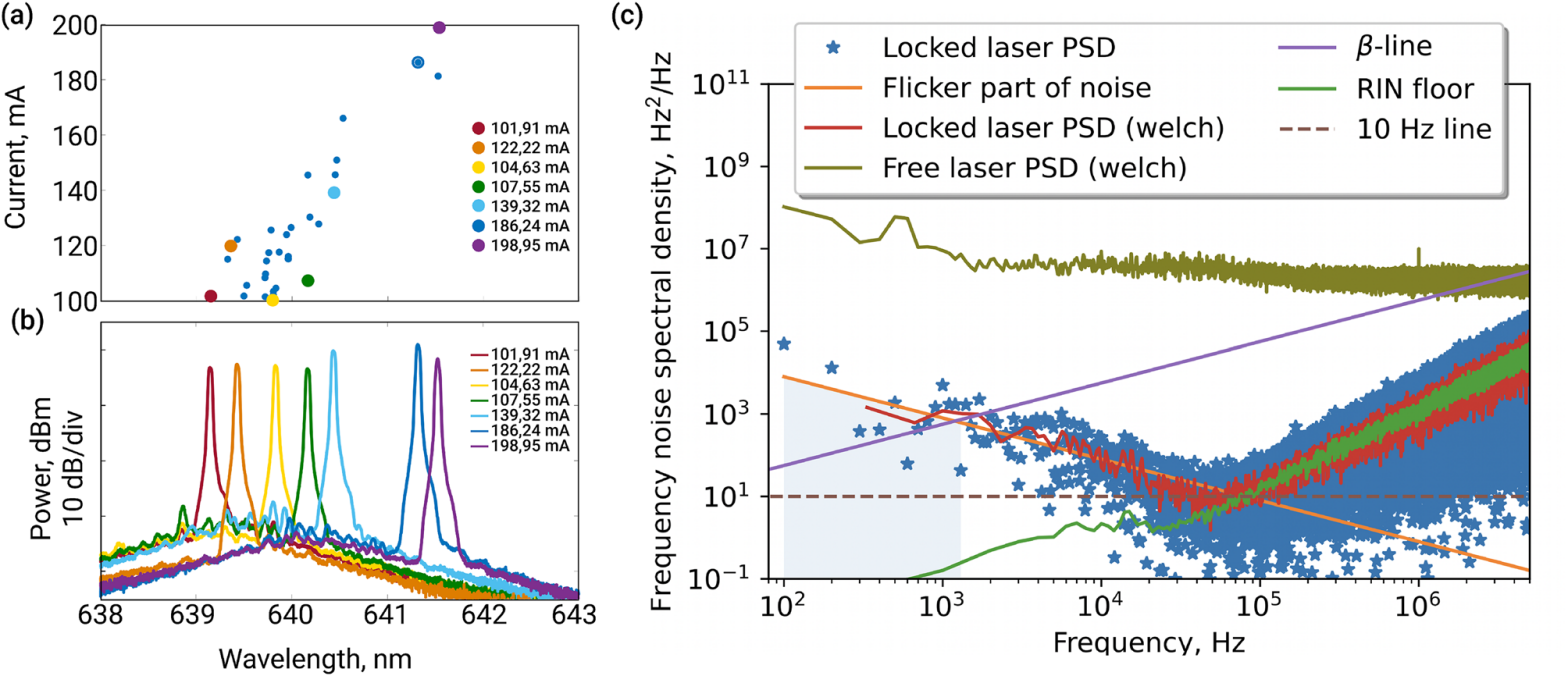
(**a**) 34 self-injection locked states for laser injection current variation from 100 to 200 mA. Laser diode frequency experiences locking to different WGMs during current increasing. (**b**) Several spectra of the self-injection locked laser diode for these selected from (**a**) injection currents. (**c**) Frequency noise spectral density of the beatnote signal of two self-injection locked laser diodes and unlocked laser (green line). The linewidth for lasers beatnote measured with $\beta$-separation line technique is 2.0 kHz providing an estimation of less than 1.4 kHz for one laser. At frequency offset $5\times 10^4$ the minimum of the frequency noise appeared at the level of an oscillator with $\approx$ 10 Hz linewidth, that characterize instantaneous linewidth. For the higher offsets, the intensity noise of the measuring equipment is dominant.

Fabry–Pérot laser diodes are spatially single-mode and have a multi-frequency spectrum. In the case of self-injection locking, the frequency spectrum collapses into a single narrow line via the mode competition process^62^. In that case, an eigenfrequency of the microresonator will determine the locked laser generation frequency. Gradually increasing the laser current, one may tune the resulting output wavelength within a relatively large range by means of mode hops. We note that each current corresponds to a single locking regime, and the trajectory of mode hops is repeatable for a given diode and temperature. Using this approach, we observe several sustainable locked states from 639 to 642 nm at 30 $^{\circ }C$ (see Fig. 2a,b). We vary the operating current from 100 to 200 mA. In Fig. 2a, 34 injection currents at which SIL was observed are presented. Seven states are highlighted with colors and presented in Fig. 2b. For higher operating current, the SIL is not achieved. It may be caused by the nonlinear deformation of the tuning curve^63^ or the nonlinear interaction between forward and backward propagating waves in the resonator^64^. It was demonstrated that the backscattering wave experiences a higher frequency shift than the forward wave due to cross-phase modulation instead of self-phase modulation caused by Kerr nonlinearity^64^.

For the temperature 20 $^{\circ }C$, the sustainable locked state can be observed in the range from 637 to 639 nm. Output power in the self-injection locking regime is observed to be almost at the same level as in the unlocked state. We measure 85 mW before locking and 80 mW after locking, corresponding to more than 90% of the total radiation. Such comparatively high output power is important for various up-to-date applications. In our case, we have relatively low efficiency of the mode excitation due to the low confinement between incident light and a WGM in terms of spot matching. So, in the case of critical coupling, the excitation efficiency remains from 10 to 30 % providing high output power.

A heterodyne method is used for the characterization of the linewidth of the stabilized lasers. There is a shortage of ultranarrow-linewidth lasers for reference, especially in the visible range. Using two equivalent laser sources in the heterodyne method allows us to overcome the limitations of the reference lasers and precisely observe spectral characteristics corresponding to the lasers under study. We lock two laser diodes to two different microresonators and using the option of the mode-hop frequency tuning to find modes having frequency difference in the bandwidth of ESA. The best results are obtained with microresonators of 3 and 4 mm diameter. We use critically coupled microresonators during the experiment, i.e., when internal losses are equal to those caused by the coupling. This condition corresponds to the maximum locking range. We collected the beatnote data of these two self-injection locked lasers with the ESA using the quadrature analysis option. Then we calculated the frequency noise spectral density of the beatnote signal (see Fig. 2c) and performed $\beta$-separation line analysis^58^ which showed a linewidth of 2.0 kHz. This value characterizes the stability of the beatnote frequency for 10 ms averaging time. In the case of two equally stable lasers, it means that the linewidth of one of them is better than $2.0/\sqrt{2} \approx 1.4\, \textrm{kHz}$. Blue dots correspond to the calculated frequency noise spectral density from the raw data. The red line in panel (c) Fig. 2 represents Welch averaging of these data. Dark green light corresponds to Welch averaging of the free-running laser frequency noise, which linewidth can be estimated as several MHz. The bright emerald line corresponds to the part of the frequency noise caused by the intensity noise. The linewidth for lasers beatnote measured with $\beta$-separation line technique is 2 kHz, where the slope of the flicker part of frequency noise density is estimated via Welch’s method of spectral density estimation^65^. Moreover, it can be seen that at the offset of $10^5$ Hz the intensity noise becomes a dominant limiting factor (see Fig. 2c, emerald green line). At the lowest point the frequency noise is better than of an effective 10-Hz-linewidth oscillator, providing an estimation of the laser instantaneous linewidth.

**Figure 3:**
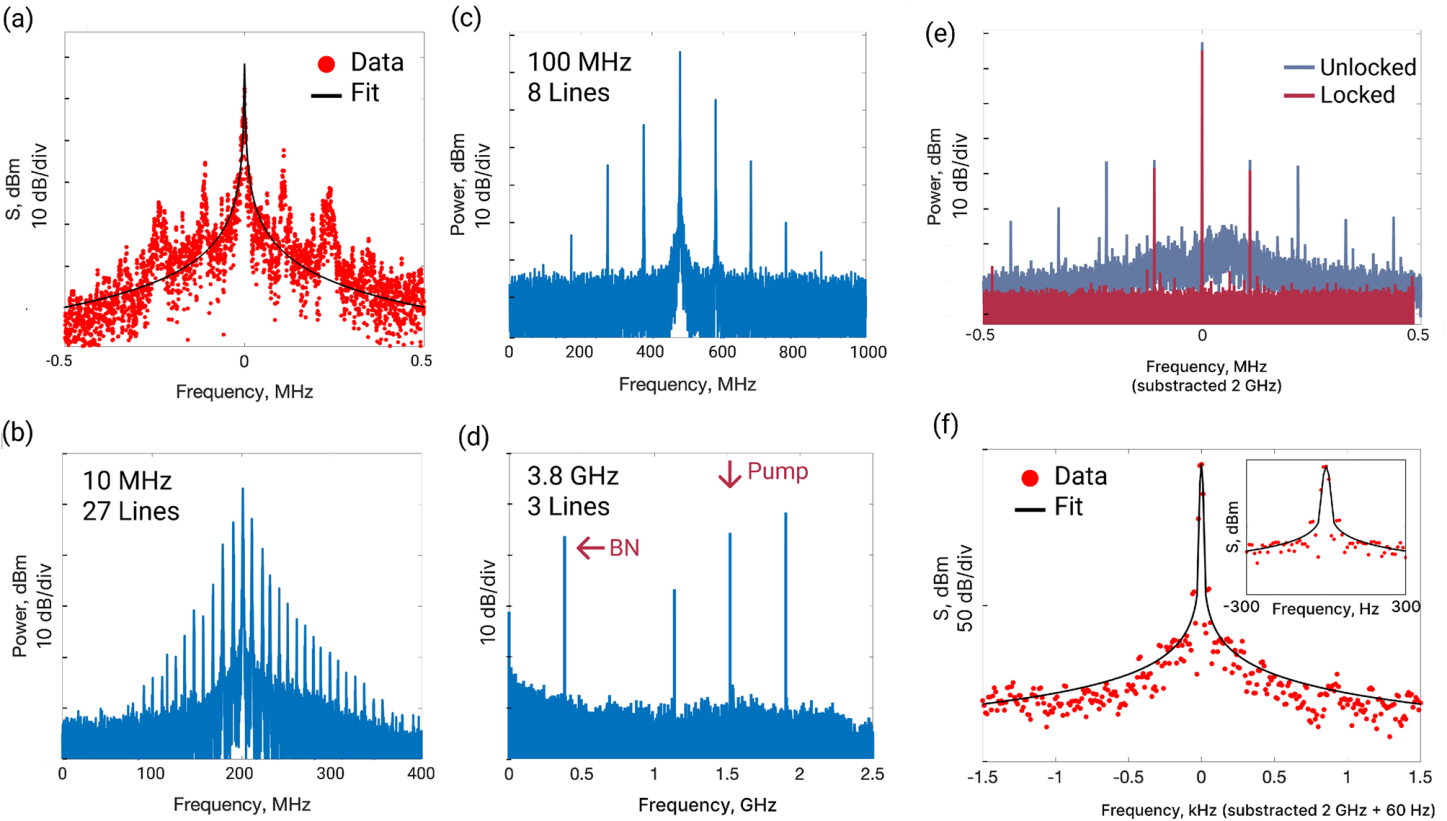
(**a**) Power spectral density of the beatnote of two SIL lasers. (**b**–**d**) Spectra of the gain-switching frequency combs for modulation frequencies (**b**) 10 MHz, (**c**) 100 MHz, (**d**) 3.8 GHz. (**e**,**f**) Power spectral density of the beatnote of the frequency comb. (**e**) Comparison between beatnotes in the free-running regime (blue) and SIL regime (red). (**f**) Voigt fit of the beatnote data. The Lorentzian part of the linewidth is 0.01 Hz, and the Gaussian part is 7 Hz at the resolution bandwidth of 10 Hz. In the inset the central part of the beatnote and approximation are presented.

The beatnote approximation gives us the Lorentzian linewidth of 1.2 kHz at the resolution bandwidth of 1 kHz and measurement time of 12.6 ms (see Fig. 3a). It is the value comparable with obtained from the frequency noise analysis. We also make an approximation with the Voigt profile, which gives us 1.2 kHz of the Lorentzian part and 90 Hz of the Gaussian part. Considering the equivalency of the lasers, the result linewidth of the lasers can be estimated as better than $1.2/\sqrt{2} = 850$ Hz.

For instance, the decent stability at ms-scale is mainly connected with acoustic insulation and thermal stabilization^66^. Some reported results in the visible band stipulate the limitations associated with the measurement technique, for example, the reference laser^5,34^. We circumvent these limitations by assembling two equivalent setups, which is as primitive as it is effective in unlocking the full potential of SIL. We demonstrate the instantaneous linewidth superior to those obtained with on-chip microresonators using the self-injection locking technique^5,35,36^ or Brillouin technique^38^ in the visible band. This may be due to the high quality factor relative to integral structures (> $10^8$).

### Gain-switched frequency combs

In previous sections, we demonstrate visible laser sources with ultra-narrow generation linewidth. It is well-known that frequency comb generation at strong normal group velocity dispersion, as is the case in the visible range, is a challenging task. To overcome that obstacle, we apply gain-switching to a SIL laser diode. It allows to generate a high-contrast frequency comb with linespacing from MHz to GHz and provides an opportunity to tune the frequency of the sidebands in the GHz scale. The tunability of the frequency interval between the lines provides a possibility of frequency scanning for several GHz in the case of self-injection locking. Operating current applies through a bias tee element and leads to the formation of the optical frequency comb. Its linespacing is equal to the modulation frequency and can be varied continuously^40^, which can be used as a frequency scan, for example, for spectroscopy. We apply 1.5 V modulation amplitude with different frequencies from 10 MHz to several GHz and achieve a gain-switching with a Fabry–Pérot laser diode. We observed spectrograms of beating signals of the SIL laser with the reference in the case of the gain-switching SIL regime. Initially, plain SIL realizes, and then gain-switching appears. It can be seen that the generation frequency remains almost unchanged during the current sweep (see Fig. 1c). Frequency tuning of linespacing can be achieved for several dozens of MHz. In Fig. 3, spectra of the beatnotes for different cases are presented. Panel (a) shows a beatnote of two SIL lasers in unmodulated case; panel (b) is a beatnote spectrum of the modulated with 10 MHz frequency laser with reference tunable laser, 27 lines are observed; panel (c) is a beatnote spectrum of the modulated with 100 MHz frequency laser with reference tunable laser, 8 lines are observed; panel (d) is a beatnote spectrum of the modulated with 3.8 GHz frequency laser with reference tunable laser, 3 lines, and 3.8 GHz interference are observed. Asymmetric power distribution can be caused by the Bogatov effect in SIL laser^62,67^.

It can be seen in Fig. 3 that SIL provides line narrowing of each comb line to the sub-kHz level. The linewidth of the beatnote of the gain-switching comb is narrow. It experiences further constriction in the SIL regime (see Fig. 3e). The noise pedestal clearly observed in the unlocked state is suppressed in the SIL state (see the red line in Fig. 3e). Comparing with results reported in Ref.^68^ where the narrowing of the beatnote signal from 100 to 50 Hz due to the self-injection locking was demonstrated in SIL GS regime, here we have the more pronounced beatnote purification up to 0.01 Hz for the Lorentzian part and 7 Hz for the Gaussian part in the Voigt profile approximation at the resolution bandwidth of 10 Hz and measurement time of 419 ms (see Fig. 3f). In the inset the same beatnote is presented in enlarged scale. The linewidth of the beatnote is comparable to the beatnotes obtained with electro-optic frequency combs^69^ and combs obtained in lithium niobate microresonators^70^. Such effect of beatnote spectral purification provides the possibility to obtain an ultrastable radio-frequency signal. It should be noted that the application of a Fabry–Pérot laser diode expands the gain-switched comb generation to more powerful laser sources up to dozens of milliwatts, compared to previously demonstrated with distributed feedback (DFB) lasers^40^.

## Conclusion

To sum up, we demonstrated the ultranarrow-linewidth lasing of the Fabry–Pérot laser diode at 638 nm obtained due to the self-injection locking to a high-Q WGM microresonator. For the characterization of the stabilized laser source, we used the heterodyne method measuring the beatnote of two equivalent SIL lasers. The instantaneous linewidth is shown to be better than 10 Hz at the averaging time of 20 $\upmu$s. The $\beta$-separation line technique provides 1.4 kHz linewidth from the frequency noise spectral density, which characterizes the stability at 10 ms measurement time. That was proved by direct observation of the beatnote of two SIL lasers at the measurement time of 12.6 ms with measured linewidth of 1.2 kHz. Relatively low efficiency of the WGM excitation during SIL allows us a concentration of more than 90 % of the total power of the free-running multifrequency regime ($\approx$ 80 mW for the studied regimes) in a locked narrow-line single-frequency lasing state. Demonstrated results are among the best in the area concerning the visible spectral range. As the next step, we demonstrated the gain-switch regime of the Fabry–Pérot laser diode. SIL provides line narrowing of each comb line to sub-kHz level and frequency combs with interline from 10 MHz to 3.8 GHz. It expands possible applications for gain-switched frequency combs in the SIL regime into the visible range and also for using Fabry–Pérot laser diodes for gain-switching. This fact demonstrates that firstly the spectrum collapse of the laser diode appears, and it becomes single-frequency. Secondly, through the gain-switching one narrow line evolves into a comb from a SIL seed. This result may be of special interest for spectroscopy, biosensing, quantum communications, atom and ion cooling, optical clocks, and other cutting-edge applications. Also, gain-switching frequency combs allow for obtaining ultra-stable and ultra-narrow RF beatnote, which is transmitted over optical fiber.

## Methods

Microresonators were made by single-point diamond turning on a lathe machine^71^. To achieve high Q-factor, the surface was polished with diamond slurries, which grain size was gradually reduced from micrometers to less than 30 nm.

We used the ringdown technique to measure the Q-factor at telecommunication wavelength^72^. The WGM was excited with a laser source, which was rapidly swept. The sweeping is faster than the decay rate of the microresonator, so when the laser stops exciting the mode we observe interference between the light from the microresonator at WGM frequency and the detuned laser. The bigger the frequency difference between the pump and eigenfrequency of the microresonator, the more frequent fringes appear in the decay. The ringdown time is proportional to the Q-factor of the mode.

In the visible band, we used a Q-factor measurement technique based on dependence of the locking width on the gap between the microresonator and a coupling prism^12^. It is based on the dependence of the linewidth of the microresonator due to the coupling losses on the distance to the coupler^61^ and the fact that the locking width is maximized at critical coupling^73^. This technique, used as a tool, provides an opportunity to measure Q-factor directly in the SIL regime. The determining of zero gap can be improved by controlling the interference pattern between the coupler and microresonator^74^, which is especially convenient for visible band.

According to $\beta$-separation line technique, the power spectral density of frequency noise $S_{\delta \nu }(f)$ can be separated into two regions. The first region contributes to the full width half maximum (FWHM) of the laser line shape. The second region contributes only to the wings of the line shape. The $\beta$-separation line determines the separation point of the two regions. The linewidth approximation formula is as follows:

1 $$\begin{aligned} FWHM = \sqrt{8\ln (2)A}, \end{aligned}$$

where A—is the area of the first region, which is formed as a region limited by the frequency noise spectral density until the crossing with the $\beta$-line, see Fig. 2c:

2 $$\begin{aligned} A = \int _{1/T_0}^\infty H(S_{\delta \nu }(f) - 8\ln (2)f/\pi ^2)S_{\delta \nu }(f) df, \end{aligned}$$

where H(x) is the Heaviside unit step function, $T_0$ is the measurement time^58^. We used Welch-averaged frequency noise as initial data for approximation for determining A in the $\beta$-line technique. The approximation was performed with 1/f dependency. The approximation is presented in Fig. 2c as a yellow line.

Welch’s method computes an estimate of the power spectral density by dividing the data into overlapping segments, computing a window tapered periodogram for each segment and averaging the periodograms, which allows to decrease variance of spectral density. The parameters of Welch’s method applied in this article are the following 50$\%$ overlapping Hamming windows with lengths of segments 30$\%$ and 10$\%$ of all data sequence lengths for locked and free running laser, respectively.

The gain-switch technique of the frequency comb generation is based on modulating the laser current up and below the lasing threshold. This can be achieved by modulating laser diode current with a large amplitude sine wave through a bias tee circuit.

## Data Availability

The datasets used and analysed during the current study available from the corresponding author on reasonable request.
